# Rethinking Blood Eosinophils for Assessing Inhaled Corticosteroids Response in COPD: A Post Hoc Analysis From the FLAME Trial

**DOI:** 10.1016/j.chest.2024.06.3790

**Published:** 2024-07-09

**Authors:** Alexander G. Mathioudakis, Sebastian Bate, Pradeesh Sivapalan, Jens-Ulrik Stæhr Jensen, Dave Singh, Jørgen Vestbo

**Affiliations:** a Division of Immunology, Immunity to Infection and Respiratory Medicine, School of Biological Sciences, The University of Manchester, Manchester, England; b North West Lung Centre, Wythenshawe Hospital, Manchester University NHS Foundation Trust, Manchester Academic Health Science Centre, Manchester, England; c Manchester Academic Health Science Centre, Research and Innovation, Manchester University NHS Foundation Trust, Manchester, England; d Centre for Biostatistics, Division of Population Health, Health Services Research and Primary Care, University of Manchester, Manchester, England; e Department of Medicine, Section of Respiratory Medicine, Copenhagen University Hospital, Herlev and Gentofte, Copenhagen, Denmark; f Department of Clinical Medicine, Faculty of Health and Medical Sciences, University of Copenhagen, Copenhagen, Denmark; g Medicines Evaluation Unit, Manchester University NHS Foundation Trust, Manchester, England

**Keywords:** blood eosinophil count, clinical trials, COPD, eosinophils, inhaled corticosteroids, precision medicine

## Abstract

**Background:**

The varied treatment response to inhaled corticosteroids (ICS) in patients with COPD and the associated increased risk of pneumonia necessitate a personalized ICS therapeutic approach. This is informed by blood eosinophil count (BEC), which predicts ICS treatment response. However, BEC appears to change in response to ICS treatment.

**Research Question:**

Does (1) BEC measured on ICS treatment (2) BEC measured off ICS treatment, or (3) the change in BEC during ICS treatment best predict treatment response to ICS in COPD?

**Study Design and Methods:**

The Fluticasone Salmeterol on COPD Exacerbations Trial (FLAME), a 52-week, double-blind randomized controlled trial compared long-acting beta-2 agonists (LABAs)/long-acting muscarinic antagonists (LAMAs) with LABA/ICS. Corticosteroids were prohibited during a 4-week run-in period. We chose patients previously on ICS, thereby allowing BEC before and after the run-in period to represent BEC on and off ICS, respectively. In this post hoc analysis, we revisited outcome data, exploring how the three BEC biomarkers interacted with treatment response to the ICS-containing regimen.

**Results:**

Our study showed that LABA/LAMA combination was superior, or at least noninferior, to LABA/ICS in curbing exacerbations for most FLAME participants. However, higher BEC off ICS, higher BEC on ICS, and significant BEC suppression during ICS treatment corresponded to superior response to LABA/ICS in terms of exacerbation rate, time to first exacerbation, and time to first pneumonia. In a subgroup, including 9% of participants, BEC changed significantly during ICS treatment (≥ 200 cells/μL), and higher BEC on ICS did not predict ICS treatment response. For these patients, BEC off ICS and BEC change proved more predictive. Excess pneumonia risk associated with ICS appeared to be confined to patients who do not benefit from this treatment. BEC was not predictive of treatment effects on lung function and health status.

**Interpretation:**

This exploratory analysis advocates preferentially using BEC off ICS or BEC change during ICS treatment for guiding ICS treatment decisions. BEC measured on ICS is less predictive of treatment response.

****Clinical** Trial Registration:**

ClinicalTrials.gov; No.: NCT01782326; URL: www.clinicaltrials.gov

**Take-home Points**

**Study Question:** Is (1) blood eosinophil count (BEC) measured while patients are not receiving inhaled corticosteroids (ICS), (2) BEC measured while patients are receiving ICS, or (3) change in BEC during ICS treatment (BEC on treatment – BEC off treatment) associated with treatment response to long-acting beta-2 agonist (LABA)/ICS vs LABA/long-acting muscarinic antagonist combinations?

**Results:** Higher BEC off ICS, higher BEC on ICS, and significant BEC suppression during ICS treatment corresponded to superior response to LABA/ICS in terms of preventing exacerbations and pneumonia. However, in 9% of participants, BEC changed significantly on ICS treatment. In this subgroup, higher BEC on ICS did not predict ICS treatment response.

**Interpretation:** Our findings advocate preferentially using BEC off ICS or BEC change during ICS treatment for guiding ICS treatment decisions.

COPD, a leading cause of death, disability, and chronic respiratory symptoms, is complex and heterogeneous, thus requiring a precision medicine therapeutic approach.^1^ Only patients with enhanced eosinophilic inflammation in the airways appear to respond to inhaled corticosteroids (ICS).^2^^,^^3^ Blood eosinophil count (BEC), a practical surrogate marker for airway eosinophilic inflammation,^2^ has been shown to predict ICS treatment response in randomized controlled trials (RCTs)^4^, ^5^, ^6^, ^7^, ^8^; higher BEC at the start of the study is associated with greater benefits for ICS treatment on exacerbations, health status, and pulmonary function.^2^^,^^9^ Because ICS may cause pneumonia and other side effects, BECs require a targeted ICS administration strategy in patients with COPD with increased exacerbation risk.^9^^,^^10^

Systemic corticosteroids suppress BEC in patients with COPD.^3^^,^^11^ In patients with asthma, it has been demonstrated that ICS can also suppress BEC.^12^^,^^13^ The potential suppression of BEC by ICS in patients with COPD could weaken the correlation between BEC and ICS treatment response. In parallel, BEC is a responsive biomarker because BEC suppression during treatment with corticosteroids has been associated with treatment response.^11^ These suggest three distinct BEC biomarkers for potential prediction of ICS treatment response: BEC measured while patients are receiving ICS (BEC on ICS), BEC measured while patients are not receiving ICS (BEC off ICS), and BEC change during ICS administration (BEC on ICS − BEC off ICS). Our prior post hoc analysis of Inhaled Steroids on Obstructive Lung Disease in Europe (ISOLDE), a 3-year RCT comparing fluticasone propionate and placebo in COPD, found BEC change to be the superior predictor.^14^^,^^15^ Intriguingly, in approximately 20% of patients, ICS treatment onset was associated with a BEC increase, and, in these patients, ICS administration was associated with a detrimental effect, characterized by a surge in exacerbation rate and accelerated FEV_1_ decline. Moreover, higher BEC off ICS but lower BEC on ICS was associated with a slower FEV_1_ decline with ICS treatment. Although ISOLDE was conducted in the 1990s under different care standards and less standardized exacerbation definitions, the analysis nonetheless suggests potential drawbacks in using BEC on ICS for COPD treatment decisions.

In this post hoc analysis of the Fluticasone Salmeterol on COPD Exacerbations Trial (FLAME) trial,^16^ we further evaluate whether BEC off ICS or BEC on ICS is better associated with treatment response to the ICS-containing regimens, primarily focusing on exacerbations. We also investigated the use of BEC change as a novel biomarker of treatment response to ICS. FLAME was chosen for this analysis due to the comprehensive capture of all three BEC biomarkers in a substantial number of participants.

## Study Design and Methods

This investigator-initiated, post hoc analysis of the FLAME trial was based on a prospectively designed analysis plan submitted to the study sponsor (Novartis) via Clinical Study Data Request (www.clinicalstudydatarequest.com).

### Overview of the FLAME Trial

The FLAME trial’s methods and results have been previously published.^16^^,^^17^ In brief, FLAME (N = 3,362), a 52-week double-anonymized, noninferiority RCT, compared the effects of combining the long-acting muscarinic antagonist (LAMA) glycopyrronium and the long-acting beta-2 agonist (LABA) indacaterol, with the combination of salmeterol (LABA) and fluticasone propionate (ICS). It found the LABA/LAMA combination superior in preventing exacerbations and improving lung function and health status over the LABA/ICS combination.^16^ Prespecified analyses by baseline BEC indicated LABA/LAMA’s benefits as superior or similar to LABA/ICS, independent of BEC.^17^

### Study Population and BEC Values

Participants underwent a 4-week run-in period while inhaled and/or systemic corticosteroids were not permitted with BEC measured before and after, the latter serving as BEC off ICS. Additionally, 56.3% of participants were already on ICS on recruitment. In this post hoc analysis, we included participants on ICS at baseline, who had their last dose within 3 days prior to the first BEC measurement (BEC on ICS). We also computed BEC change (BEC on ICS minus BEC off ICS), where a negative value indicates BEC suppression during ICS treatment. We used the absolute BEC values (cells/μL) in the analyses.

### Outcomes of Interest

This post hoc analysis investigated the correlation between three prespecified BEC biomarkers and treatment response to the ICS-containing regimen. The primary outcomes were the rates of (1) moderate or severe and (2) severe exacerbations. Secondary outcomes included the rate of all exacerbations, rate of exacerbations according to their treatment (only corticosteroids, only antibiotics, or both), time to first exacerbation, time to first pneumonia, and change from baseline in St. George’s Respiratory Questionnaire, in postbronchodilator FEV_1_, and in FVC.

### Subgroup and Sensitivity Analyses

Our main analyses were reiterated for the subgroup of participants exhibiting at least a 200 cells/μL BEC change (suppression or rise, henceforth, significant BEC change). This threshold was selected by consensus among the investigators, and the aim of this analysis was to exclude smaller differences in BEC that could be driven by random variability of the biomarker.^18^ We conducted head-to-head comparisons of LABA/LAMA and LABA/ICS in specific subgroups, including patients with high BEC off ICS (≥ 200 cells/μL) but low BEC on ICS (< 200 cells/μL) and vice versa, and those showing significant BEC suppression (≥ 200 cells/μL) vs those without.

In a sensitivity analysis, we only incorporated participants who were administered an ICS dose within the 2 days preceding the BEC on ICS measurement.

### Statistical Analyses

We used appropriate statistical models to examine the interaction between treatment and BEC biomarkers. For the rate of exacerbations, we used a generalized linear model with a negative binomial distribution and included a time on treatment offset. The impact of treatment on time to first exacerbation, pneumonia, or death was assessed via Cox proportional hazards model. We verified the proportional hazards assumption using Kaplan-Meier curves and the Schoenfeld residuals.

Furthermore, mixed-effect model repeated measures were used to evaluate treatment effects on change in pulmonary function and health status, focusing on the three-way interaction between treatment, time, and BEC parameters.

We adjusted all analyses for age, sex, smoking status, prior exacerbation history, prior LABA or LAMA use, baseline COPD assessment test score, and baseline FEV_1_. With < 1% missing values for each parameter, there were no significant gaps in any covariate. All analyses were conducted in R v.3.6.3 (R Core Team).

## Results

The post hoc analysis inclusion criteria were fulfilled by 1,332 FLAME trial participants (39.6%), split almost evenly between those receiving LABA/LAMA and LABA/ICS combinations. Baseline characteristics were similar between groups (Table 1).

**Table 1:** Baseline Characteristics of the Participants Included in This Post Hoc Analysis

Characteristic	LABA/LAMA (n = 673)	LABA/ICS (n = 659)
Age, y	64.1 ± 8.0	64.3 ± 7.8
Female sex	180 (26.7)	192 (29.1)
Active tobacco use	241 (35.8)	240 (36.4)
COPD severity, spirometric stages		
GOLD I – mild	0 (0)	0 (0)
GOLD II – moderate	206 (30.6)	179 (27.2)
GOLD III – severe	406 (60.3)	422 (64.0)
GOLD IV – very severe	56 (8.3)	54 (8.2)
Baseline treatment		
LABA	586 (87.1)	597 (90.6)
LAMA	389 (57.8)	395 (59.9)
LABA + LAMA + ICS	362 (53.8)	382 (58.0)
Postbronchodilator FEV_1_, % predicted	43.7 (9.4)	43.4 (9.3)
Postbronchodilator ratio of FEV_1_ to FVC, %	41.1 (9.5)	41.4 (9.8)
Exacerbations during the preceding year		
1	533 (79.2)	510 (77.4)
≥ 2	140 (20.8)	149 (22.6)

Values are mean ± SD or No. (%). GOLD = Global Initiative for Chronic Obstructive Lung Disease; ICS = inhaled corticosteroids; LABA = long-acting beta-2 agonist; LAMA = long-acting muscarinic antagonist.

### Comparison of BEC Off ICS and BEC On ICS

#### Exacerbations

**Figure 1:**
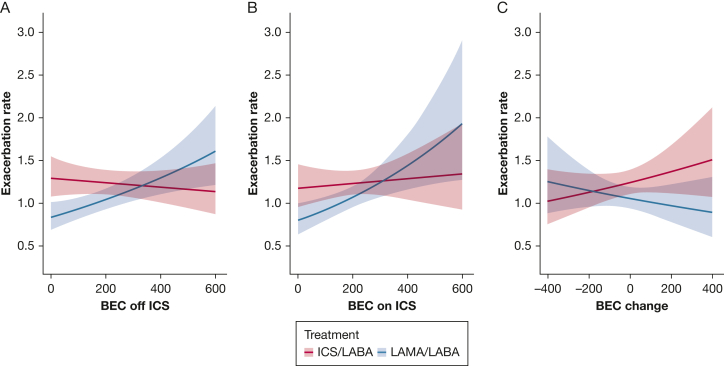
Impact of LABA/LAMA vs LABA/ICS on the frequency of moderate or severe exacerbations according to BEC off ICS, BEC on ICS, and BEC change. BEC = blood eosinophil count; ICS = inhaled corticosteroids; LABA = long-acting beta-2 agonist; LAMA = long-acting muscarinic antagonist.

Approximately one-half of the eligible participants (47.9%) experienced at least one moderate or severe exacerbation during study follow-up. BEC off ICS significantly interacted with treatment effect on the rate of moderate or severe exacerbations (*P* for interaction < .001). Higher BEC off ICS was related to a superior response to LABA/ICS (intersection point: 340 cells/μL) (e-Table 1, Fig 1). The model based on BEC on ICS was similar to the one based on BEC off ICS, but the interaction term did not reach statistical significance (*P* = .069). In general, the CIs for both analyses did not clearly separate due to the variability in treatment effect. Both higher BEC off and on ICS were associated with better LABA/ICS response in terms of the time to first moderate or severe exacerbation (*P* < .001 and *P* < .008, respectively) (e-Table 2).

Neither BEC off ICS nor BEC on ICS demonstrated a substantial interaction with treatment effect on the rate or time to first severe exacerbation, perhaps due to a low event count, with only 139 participants experiencing severe exacerbations (e-Fig 1, e-Tables 1, 2).

**Figure 2:**
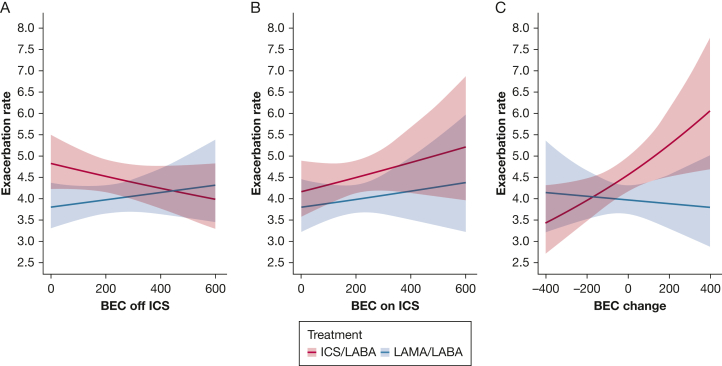
Impact of LABA/LAMA vs LABA/ICS on the frequency of any (mild, moderate, or severe) exacerbations according to BEC off ICS, BEC on ICS, and BEC change. BEC = blood eosinophil count; ICS = inhaled corticosteroids; LABA = long-acting beta-2 agonist; LAMA = long-acting muscarinic antagonist.

The association between BEC off ICS and treatment response on the rate of exacerbations of any severity (mild, moderate, or severe) did not reach statistical significance (*P* = .089), but there was an association between higher BEC off ICS and favorable treatment response to LABA/ICS on the time to first exacerbation of any severity (*P* = .006) (e-Tables 1, 2, Fig 2). BEC on ICS was not associated with either of these outcomes (*P* = .886 and *P* = .109, respectively).

Analyses by exacerbation subtype based on treatment received (oral corticosteroids and/or antibiotics) are presented in e-Tables 1 and 2 and e-Figures 2-4; analyses evaluating exacerbations treated with systemic corticosteroids but not antibiotics showed results that were consistent with the overall analysis of moderate or severe exacerbations.

#### Pneumonia

BEC off ICS, but not BEC on ICS, was associated with treatment effect on time to first pneumonia (*P* = .041 and *P* = .467, respectively) (e-Table 3). These associations were based on a small number of participants experiencing pneumonia during the study follow-up (n = 44). According to our model, the annual risk of pneumonia among FLAME participants with a BEC off ICS of 100 cells/μL receiving LABA/ICS was 5.3%, compared with 1.8% among those receiving LABA/LAMA. The corresponding percentages for participants with BEC off ICS of 400 cells/μL were 3.9% and 3.45%, respectively.

#### Other Outcomes

Neither BEC off ICS nor BEC on ICS were predictive of treatment response regarding the change from baseline in pulmonary function (FEV_1_ or FVC) or health status (St. George’s Respiratory Questionnaire).

### BEC Change (BEC On Minus BEC Off ICS)

**Figure 3:**
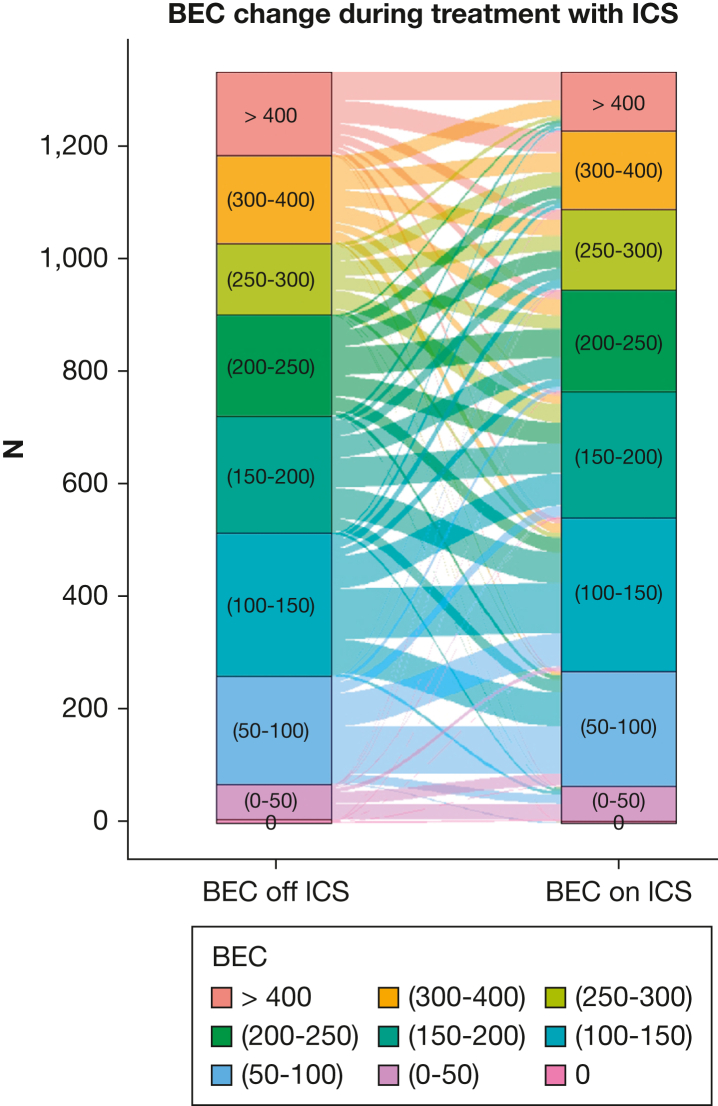
Alluvial diagram depicting BEC off ICS, BEC on ICS, and BEC change during treatment with ICS among the study participants. BEC = blood eosinophil count; ICS = inhaled corticosteroids.

A BEC change threshold of > 50 cells/μL was selected for comparing measurements on vs off ICS because it approximates the minimum discriminatory difference for blood eosinophils, accounting for limitations in reproducibility.^19^ Based on this threshold, BEC on ICS was higher in 19.5%, remained unchanged in 55.2%, and decreased in 25.3% of participants (e-Fig 5, Fig 3). From 738 participants with BEC off ICS < 200 cells/μL, 18% showed higher values (≥ 200 cells/μL) while on ICS. Among 572 participants with BEC off ICS ≥ 200 cells/μL, 30.6% had BEC on ICS < 200 cells/μL. BEC on ICS was significantly lower than BEC off ICS (median difference, –10 cells/μL; *P* < .001).

### BEC Change as a Predictive Therapeutic Biomarker

BEC change significantly interacted with treatment effect on the rate of moderate or severe exacerbations (*P* = .036), rate of exacerbations of any severity (*P* = .041), time to first moderate or severe exacerbation (*P* = .011), and time to first pneumonia (*P* = .049) (e-Tables 1-3; Figure 2, Figure 3). BEC rise during ICS treatment was associated with an inferior response to LABA/ICS, whereas significant BEC suppression indicated an opposite trend. In our primary outcome (rate of moderate or severe exacerbations), the intersection point where LABA/ICS appeared to be equally effective with LABA/LAMA was observed at −170 cells/μL.

**Figure 4:**
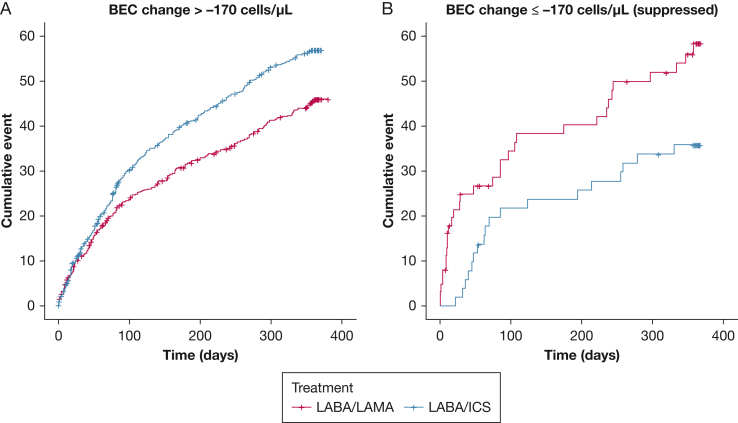
A-B, Time to first moderate or severe exacerbation stratified by treatment (LABA/LAMA vs LABA/ICS) among participants with (A) BEC change > −170 cells/μL and (B) BEC change ≤ −170 cells/μL (significantly suppressed). The opposite treatment effects observed between patients with significantly suppressed BEC and those without should be noted. BEC = blood eosinophil count; ICS = inhaled corticosteroids; LABA = long-acting beta-2 agonist; LAMA = long-acting muscarinic antagonist.

**Figure 5:**
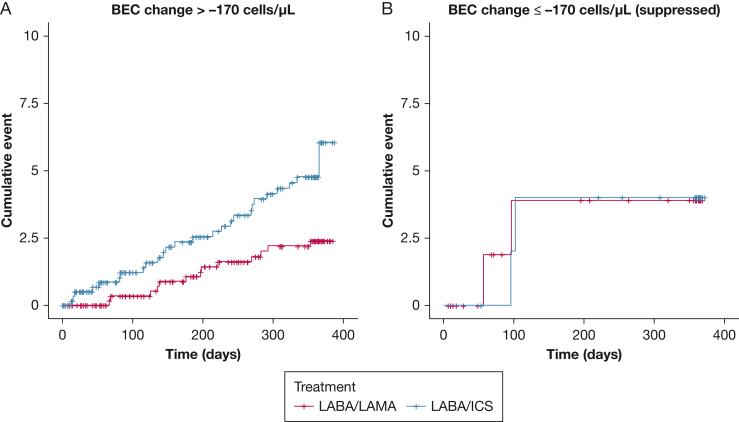
A-B, Time to first episode of pneumonia stratified by treatment (LABA/LAMA vs LABA/ICS) among participants with (A) BEC change > −170 cells/μL and (B) BEC change ≤ −170 cells/μL (significantly suppressed). The opposite treatment effects observed between patients with significantly suppressed BEC and those without should be noted. BEC = blood eosinophil count; ICS = inhaled corticosteroids; LABA = long-acting beta-2 agonist; LAMA = long-acting muscarinic antagonist.

**Figure 6:**
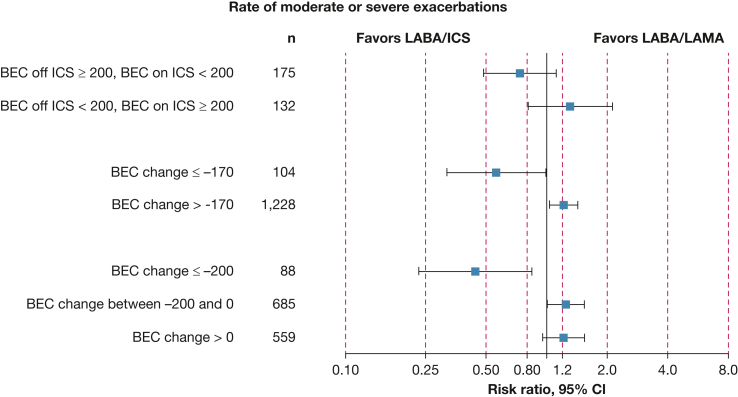
Impact of LABA/LAMA vs LABA/ICS on the rate of moderate or severe exacerbations in subgroup of patients with diverging BEC off vs on ICS. OR < 1 favors LABA/ICS. BEC = blood eosinophil count; ICS = inhaled corticosteroids; LABA = long-acting beta-2 agonist; LAMA = long-acting muscarinic antagonist.

We grouped participants based on BEC change at the identified threshold of −170 cells/μL (e-Figs 6-12, Figure 4, Figure 5, Figure 6). Notably, LABA/ICS was superior in reducing the rate of moderate or severe exacerbations among 104 participants (7.8%) with BEC suppression of at least 170 cells/μL (OR, 0.56; 95% CI, 0.32-0.98; *P* = .022), whereas the opposite effect was observed among the 1,228 participants (92.1%) with higher BEC change (> −170 cells/μL; rate ratio, 1.21; 95% CI, 1.03-1.43; *P* < .001). Similar trends were observed in other exacerbation outcomes and pneumonia. We also evaluated the rate of moderate or severe exacerbations using BEC change of ≤ −200, −200 to 0, and > 0 cells/μL, and observed that LABA/ICS was the superior treatment in the former group, whereas LABA/LAMA was superior in the last two groups (Fig 6).

We evaluated the subgroup of 120 participants (9.0%) where BEC off ICS vs BEC on ICS differed significantly, by at least 200 cells/μL (e-Tables 4-6, e-Figs 13-20). BEC suppression was observed in 88 of these participants, and BEC rise was observed in 32. In this subgroup, higher BEC off ICS and BEC suppression both predicted improved ICS response in the rate of moderate or severe exacerbations, time to first moderate or severe exacerbation, and time to first pneumonia. The interaction between treatment response and BEC on ICS was significant only for the rate of exacerbations treated with systemic corticosteroids alone. Visually, in this subgroup, lower BEC on ICS appeared to be associated with LABA/ICS superiority (e-Figs 13-15). In the complementary set of participants where BEC off ICS vs BEC on ICS differed by < 200 cells/μL, the association of both BEC off ISC and BEC on ICS with treatment effects on the rate of moderate or severe exacerbations was similar to the main analysis (e-Fig 16).

Within the subgroup of 175 participants (13.1%) who had BEC off ICS ≥ 200 cells/μL with BEC on ICS below this threshold, there was a trend for a reduced rate of moderate or severe exacerbations favoring LABA/ICS (rate ratio, 0.74; 95% CI, 0.49-1.12) (Fig 6). The opposite trend was observed among 132 participants (9.9%) with BEC off ICS < 200 cells/μL and BEC on ICS above this threshold (rate ratio, 1.31; 95% CI, 0.81-2.12). Similar trends were observed in the respective subgroup analyses of the remaining exacerbation and pneumonia outcomes (e-Figs 11-12).

BEC change was not predictive of treatment response regarding the change from baseline in pulmonary function or health status.

### Sensitivity Analysis

In the subgroup of 446 participants (33.5%) who had an ICS dose within 2 days prior to BEC on ICS measurement, most results were not statistically significant, likely due to the smaller sample size. Reassuringly, potential association directions were generally consistent with primary analyses (e-Figs 21-28. e-Tables 7-9).

## Discussion

This post hoc analysis of the FLAME trial has elucidated important findings relevant to the use of BEC as a biomarker to personalize ICS treatment strategies in COPD. In the FLAME subgroup analyzed, LABA/LAMA combination was superior or at least noninferior to LABA/ICS in preventing exacerbations in most of the participants, consistent with the previously published results in the overall population.^20^ Higher BEC off ICS and BEC on ICS are generally correlated with a greater risk reduction of exacerbations with ICS treatment. However, in 9% of study participants, BEC changed significantly during ICS treatment, and in these patients, higher BEC on ICS did not necessarily predict a favorable response to LABA/ICS. In parallel, we assessed BEC change during ICS treatment, as a continuous variable, as a potential novel biomarker; the FLAME dataset is unique in allowing the relationship between BEC change and treatment effect to be investigated. We show that considerable suppression of BEC may occur in some individuals, which is associated with a greater ICS treatment effect. In contrast, lack of BEC suppression or an increase in BEC was a predictor of a better response to LAMA/LABA. These results highlight the potential utility of BEC change during ICS treatment as a predictive biomarker of treatment response to ICS in COPD. Finally, BEC off ICS and BEC change findings suggested that the excess pneumonia risk associated with ICS is confined to patients who do not benefit from this treatment.

Several studies have demonstrated a variability of BEC in COPD,^18^^,^^21^ which is greater at higher BEC. An unresolved question has been whether ICS treatment can change BEC. We show that the overall change is small (−10 cells/μL), but considerable change was observed in some individuals (eg, 9% had change ≥ 200 cells/μL). BEC suppression was associated with greater ICS response in different analyses, with BEC change −170 cells/μL appearing to split the population neatly into a group favoring LABA/ICS (n = 104) and a group favoring LAMA/LABA treatment (n = 1,228). Clinical trial populations with higher exacerbation risk (compared with FLAME) have shown a greater benefit for LABA/ICS over LAMA/LABA; therefore, the threshold reported here will likely vary in other populations.^4^^,^^5^ Nevertheless, the current analysis demonstrates that ICS can suppress BEC in COPD, and that the ICS-related BEC change can be used to predict ICS responses.

BEC change suggests that BEC on ICS is not equivalent to BEC off ICS. Furthermore, the BEC change results imply that BEC on ICS may misclassify some patients (9% of this study participants) regarding ICS response prediction (eg, higher BEC on ICS plus lower BEC off ICS seems to predict ICS nonresponse, but using only [higher] BEC on ICS in this individual may mistakenly predict a positive ICS response and vice versa). This can explain why BEC on ICS had less clear separation of CIs for moderate to severe exacerbations compared with BEC off ICS (Fig 2) and performed less well for some clinical outcomes compared with BEC off ICS (eg, frequency of any exacerbation, time to first pneumonia). The design of previous RCTs could explain why these BECs on ICS signals have been missed so far (discussion in supplement 5).

**Figure 7:**
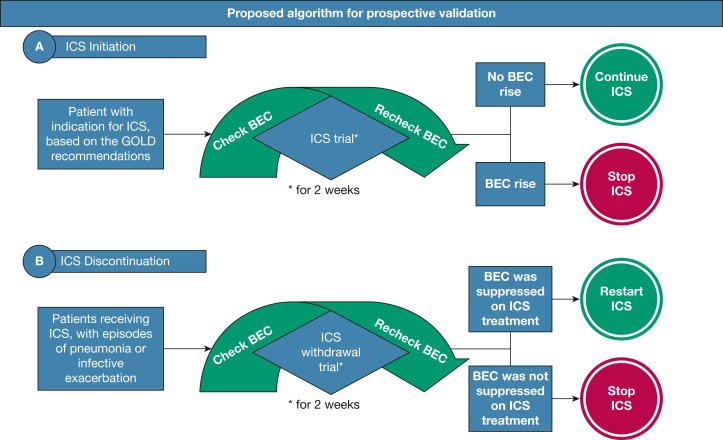
A, B, Proposed simple algorithm for ICS (A) initiation or (B) discontinuation based on the findings of this post hoc analysis. These findings should be validated prospectively prior to informing clinical practice. BEC = blood eosinophil count; GOLD = Global Initiative for Chronic Obstructive Lung Disease; ICS = inhaled corticosteroids.

Our findings, although requiring prospective validation, reveal important potential implications for clinical practice. First, BEC change is a new biomarker that appears to have utility and may explain ICS nonresponse in some individuals with higher BEC on ICS. Second, BEC on and BEC off ICS are not identical, with BEC off ICS appearing to offer some advantages in the prediction models performed. European Respiratory Society guidelines^22^ on ICS withdrawal in COPD recommend using BEC measured on treatment for making decisions around ICS withdrawal; our findings suggest some caution. If BEC change is used, a relevant question is how to handle individuals without BEC change. Although BEC change −200 cells/μL to 0 cells/μL appeared to favor LAMA/LABA (Fig 6), there is likely a heterogeneous response within this group that is related to the absolute BEC, and a subanalysis of individuals with < 200 cells/μL change showed that both BEC off ICS and BEC on ICS enabled prediction in this subgroup. Putting all these together reveals the following: First, for the ICS-naïve patients, ICS should be offered for patients with Global Initiative for Chronic Obstructive Lung Disease group E with ongoing eosinophilic inflammation and exacerbations, in line with Global Initiative for Chronic Obstructive Lung Disease recommendations; however, a repeat, on-treatment BEC could further inform clinical decisions, with a low threshold for discontinuing in case of BEC rise. Second, history of infective exacerbations or episodes of pneumonia may be a more appropriate indication for considering ICS withdrawal compared with BEC measured on treatment. BEC measured on ICS treatment and during a short trial of ICS withdrawal could guide treatment decisions (Fig 7).

Our analysis also demonstrated a strong association between BEC biomarkers and pneumonia risk among patients with COPD receiving ICS. The heightened pneumonia risk was primarily observed in patients unlikely to benefit from ICS. This aligns with past research indicating susceptibility to bacterial infections in noneosinophilic COPD^23^, ^24^, ^25^ and reinforces the need to target ICS use to avoid unnecessary risks. We did not establish a link between BEC variables and treatment effect on pulmonary function and health status trajectories. This could be due to the short follow-up period of 1 year, which might not be sufficient to evaluate treatment impact on pulmonary function decline rate. Furthermore, lack of between-treatment differences is not surprising because dual bronchodilators are known for their excellent activity on these outcomes.^26^

A limitation is that the impact of ICS on BEC may have been underestimated in this study. In FLAME, BEC on ICS and bronchodilator reversibility were tested at the run-in visit and, in preparation of the latter, 66.5% of these post hoc study participants did not receive ICS for 3 days prior to BEC measurement. Although the exact timeline of BEC response to the initiation or discontinuation of ICS is uncertain, indirect data from the Eosinophil-guided Corticosteroid Therapy in Patients Admitted to Hospital With COPD Exacerbation (CORTICO-COP) trial suggest effects start diminishing within 48 h.^27^ Therefore, in our analysis, ICS impact on BEC had likely began to diminish when BEC on ICS was measured. Indeed, in the ISOLDE post hoc analysis, which did not have a similar limitation, an inverse association was observed between BEC on ICS and treatment response to ICS, across the whole study population.^14^ In general, the ISOLDE post hoc analysis strongly supports the main findings of this analysis (details available in supplement 6).

Another important limitation of this study is its post hoc, exploratory nature that means the results should be interpreted with caution and validated prospectively. However, this analysis was based on a prospectively developed protocol that was submitted to the study sponsor prior to accessing the study data. Furthermore, FLAME compared LABA/LAMA with LABA/ICS; the latter combination is not recommended for COPD management anymore. However, > 50% of the study participants were receiving triple combination (LABA/LAMA/ICS) prior to recruitment, and for this reason we think these findings are generalizable, especially the value of BEC change and observations regarding the association between BEC and ICS withdrawal. Moreover, we cannot exclude the possibility that some of the participants may have had concomitant, undiagnosed asthma that could have impacted our findings. However, FLAME strictly excluded patients with any diagnosis of asthma (current or historic), those with respiratory symptoms onset prior to 40 years of age, those with allergic rhinitis, and those with a very high BEC (> 600 cells/μL) at baseline. These criteria are much stricter than other large RCTs. Finally, the timing of BEC measurement was not standardized, and this may have somewhat impacted our findings because BEC has a well-described circadian pattern.^28^

## Interpretation

The findings from the two post hoc analyses of the ISOLDE and FLAME trials highlight the need for a prospective study of BEC assessment timing and efficacy of ICS. They also question the use of BEC to guide withdrawal of ICS because it appears that some ICS responders may actually have low BEC on ICS and vice versa. BEC remain a predictor of ICS response, but our data adds some complexity to the way that BEC could be interpreted.

## Funding/Support

A. G. M., D. S., and J. V. were supported by the 10.13039/501100000272National Institute for Health and Care Research (NIHR) 10.13039/100014653Manchester Biomedical Research Centre [Grant NIHR203308]. A. G. M. was supported by an NIHR Clinical Lectureship in Respiratory Medicine.

## Financial/Nonfinancial Disclosures

The authors have reported to *CHEST* the following: A. G. M. reports honoraria for presenting from GSK, not related to this work. D. S. reports grants and personal fees from AstraZeneca, Boehringer Ingelheim, Chiesi, GlaxoSmithKline, Glenmark, Menarini, Mundipharma, Novartis, Pfizer, Pulmatrix, Theravance, and Verona; and personal fees from Cipla, Genentech, and Peptinnovate, not related to this work. J. V. reports honoraria for consulting and/or presenting from ALK, AstraZeneca, Boehringer Ingelheim, Chiesi, GSK, Novartis, and Teva, not related to this work. None declared (S. B., P. S., J.-U. S. J.).

## Supplementary Data

e-Online Data
